# Rate constants of chlorine atom reactions with organic molecules in aqueous solutions, an overview

**DOI:** 10.1007/s11356-022-20807-9

**Published:** 2022-06-10

**Authors:** László Wojnárovits, Jianlong Wang, Libing Chu, Erzsébet Takács

**Affiliations:** 1 Institute for Energy Security and Environmental Safety, Centre for Energy Research, Radiation Chemistry Department, ELKH, Budapest, Hungary; 2 Institute of Nuclear and New Energy Technology, Tsinghua University, Beijing, 100084 People’s Republic of China

**Keywords:** Chlorine atom, Rate constant, Organic pollutants, UV/chlorine process, Reaction mechanism

## Abstract

Rate constants of chlorine atom (Cl^•^) reactions (*k*_Cl•_) determined using a large variation of experimental methods, including transient measurements, steady-state and computation techniques, were collected from the literature and were discussed together with the reaction mechanisms. The *k*_Cl•_ values are generally in the 10^8^–10^9^ mol^−1^ dm^3^ s^−1^ range when the basic reaction between the Cl^•^ and the target molecule is H-atom abstraction. When Cl^•^ addition to double bonds dominates the interaction, the *k*_Cl•_ values are in the 1 × 10^9^–2 × 10^10^ mol^−1^ dm^3^ s^−1^ range. In the *k*_Cl•_ = 1 × 10^10^–4 × 10^10^ mol^−1^ dm^3^ s^−1^ range, single-electron-transfer reactions may also contribute to the mechanism. The Cl^•^ reactions with organic molecules in many respects are similar to those of ^•^OH, albeit Cl^•^ seems to be less selective as ^•^OH. However, there is an important difference, as opposed to Cl^•^ in the case of ^•^OH single-electron-transfer reactions have minor importance. The uncertainty of Cl^•^ rate constant determinations is much higher than those of ^•^OH. Since Cl^•^ reactions play very important role in the emerging UV/chlorine water purification technology, some standardization of the rate constant measuring techniques and more *k*_Cl•_ measurements are recommended.

## Introduction

Recently the UV/chlorine technique has been investigated as an alternative to the UV/H_2_O_2_ advanced oxidation process (AOP) and has been tested at a few water treatment utilities (Jin et al. 2011; De Laat and Stefan 2018; Zhang et al. 2018; Kishomoto 2019). The degradation of organic pollutants during the UV/chlorine process takes place by several parallel reactions, e.g., direct photolysis, if the organic compounds absorb the UV radiation used, oxidation by the free radicals (hydroxyl radical (^•^OH), chlorine atom (Cl^•^) and also other radical species). In aqueous solutions, the free chlorine species exist in three forms: at low pH the Cl_2_ form, between pH 1 and 8 the HOCl form and at high pH ClO^–^ form dominates.

1 $$\begin{array}{cc}{\mathrm{Cl}}_2+{\mathrm H}_2\mathrm O\rightleftharpoons\mathrm{HOCl}+\mathrm{HCl}&K=3.94\times10^{-4}\;\mathrm{mol}^2\;\mathrm{dm}^{-6}\end{array}$$

2 $$\mathrm{HOCl}\rightleftharpoons\mathrm H^++\mathrm{OCl}^-\left(\mathrm{equilibrium}\;\mathrm{with}\;\mathrm pK_{\mathrm a}=7.6\;\mathrm{at}\;20^\circ\mathrm C\right)$$

The UV photolysis of HOCl gives ^•^OH and Cl^•^ with quantum yields close to unity. With lower yield, Cl^•^ also forms in the photolysis of ClO^–^.

3 $$\mathrm{HOCl}+hv\to {\mathrm{Cl}}^{\bullet }+{}^{\bullet }\mathrm{OH}$$

4 $$\mathrm{OCl}+hv\to \to {}^{\bullet }{\mathrm{O}}^{-}+{\mathrm{Cl}}^{\bullet }$$

5 $$\begin{array}{cc}{}^{\bullet }{\mathrm{O}}^{-}+{\mathrm{H}}_{2}\mathrm{O}\to {}^{\bullet }\mathrm{OH}+{\mathrm{OH}}^{-}& \left(\mathrm{p}{K}_{\mathrm{a}}=11.9\right)\end{array}$$

From these equations, it is obvious that the reactions of the chlorine atom play an important role during the UV/chlorine-advanced oxidation process. In such systems, ^•^OH is always present. Based on reactions (3)–(4), equal yields of Cl^•^ and ^•^OH are expected. However, in practice the ^•^OH yield is higher at high HOCl concentrations, since Cl^•^ may abstract an H-atom from HOCl increasing by that the abundance of ^•^OH. The reactions of Cl^•^ may lead to formation of undesired chlorinated derivatives and/or promote their oxidation as observed for several aromatic molecules (Mártire et al. 2001; Lei et al. 2021), e.g., in the reaction of Cl^•^ with benzene chlorobenzene forms, albeit with low yield (Alegre et al. 2000).

Cl^•^ reactions are also very important in the atmospheric chemistry. Cl^•^ can form in the cloud droplets by the reactions of Cl^–^ with strongly oxidizing species such as NO_3_^•^, SO_4_^•–^ and ^•^OH (X^•^) (Buxton et al. 2000; Herrmann 2003). Therefore, Cl^–^ oxidation may represent an important sink of strong oxidants in the tropospheric systems.

6 $${\mathrm{Cl}}^{-}+{\mathrm{X}}^{\bullet }\to {\mathrm{Cl}}^{\bullet }+{\mathrm{X}}^{-}$$

In the presence of Cl^–^ in aqueous solutions, Cl^•^ is in equilibrium (7) with Cl_2_^•–^ with an equilibrium constant of *K* = 1.4 × 10^5^ mol^–1^ dm^3^ (Buxton et al. 1998).

7 $${\mathrm{Cl}}^{\bullet }+{\mathrm{Cl}}^{-}\rightleftharpoons {\mathrm{Cl}}_{2}^{\bullet -}$$

Because of the lack of Cl^–^, this equilibrium does not exist in organic solvents (e.g., CCl_4_). Due to the simplicity, many Cl^•^ rate constant measurements, especially in the early period of radical chemistry investigations, were carried out in non-aqueous systems (Alfassi et al. 1989; NDRL, NIST, 2022). These rate constants may differ considerably from the ones determined in aqueous system. Therefore, they are not considered in the present overview.

Equilibrium (7) is established quickly and the reactions of Cl_2_^•–^ contribute to the degradation of pollutants (Buxton et al. 2000; Mártire et al. 2001). Cl_2_^•–^ is less reactive than Cl^•^. Cl^•^ and Cl_2_^•–^ are oxidants (*E*(Cl^•^/Cl^–^) = 2.4 V and *E*(Cl_2_^•–^/2Cl^–^) = 2.1 V *vs*. NHE, Armstrong et al. 2015) and they react with many organic molecules.

### Production of Cl^•^ in aqueous solution and methods of rate constant determination

The techniques used for rate constant determination differ in many respects. In some of the techniques, Cl^–^ serves as source of Cl^•^, in others chlorine containing organic or inorganic compounds are used in Cl^•^ production (chloroacetone, chloramine).

There are two basic techniques for the determination of aqueous-phase rate constants of reactions between radicals and target molecules: the direct and the indirect methods. These techniques have been reviewed and compared in a recent paper published by Ma et al. (2021). When transient techniques (direct method), pulse radiolysis or (laser) flash photolysis are used for rate constant determination, reactive radicals are produced by the energy absorption from a short pulse of accelerated electrons, or (laser) flesh light and the time dependence of transient light absorption of the radical is detected. The *k*_Cl•_ values are determined from the time dependences of the transient absorbance signals. In steady-state experiments (indirect method), mostly relative rate constants are measured (*k*_Cl•_/*k*_competitor_) and the absolute value of the compound of interest is calculated by multiplying the relative value by the known rate constant of the competitor. Lei et al. (2019) combined the transient and competitive techniques (see later). A number of rate constants were determined in UV/chlorine process and applying scavenging experiments or complex kinetic modeling to derive *k*_Cl•_.

In Cl^–^ solutions, most often sulfate radical anions (SO_4_^•–^) were used for Cl^–^ oxidation to Cl^•^ (Eq. 8). In radiolytic reactions (Eq. (9)), SO_4_^•–^ forms in the reaction of hydrated electrons (e_aq_^–^) with persulfate anions (S_2_O_8_^2–^) (Buxton et al. 1998). In kinetic studies on Cl^•^ reactions, the photolysis of persulfate anions (Eq. (10)) was also often used to generate SO_4_^•–^ in both transient and steady-state experiments (Zhu et al. 2005; Caregnato et al. 2007; Alegre et al. 2000; Mártire et al. 2001; Ma et al. 2021). Zhu et al. (2019) in their steady-state experiments activated persulfate by Fe^2+^ ions (11), similar activations were also made using other transient metal ions (e.g., Ti(III), Gilbert et al. 1988).

8 $${\mathrm{SO}}_{4}^{\bullet -}+{\mathrm{Cl}}^{-}\to {\mathrm{SO}}_{4}^{2-}+{\mathrm{Cl}}^{\bullet }$$

9 $${{\mathrm{S}}_{2}\mathrm{O}}_{8}^{2-}+{\mathrm{e}}_{\mathrm{aq}}^{-}\to {\mathrm{SO}}_{4}^{\bullet -}+{\mathrm{SO}}_{4}^{2-}$$

10 $${{\mathrm{S}}_{2}\mathrm{O}}_{8}^{2-}+\mathrm{hv}\to {2\mathrm{SO}}_{4}^{\bullet -}$$

11 $${\mathrm{Fe}}^{2+}+{{\mathrm{S}}_{2}\mathrm{O}}_{8}^{2-}\to {\mathrm{Fe}}^{3+}+{\mathrm{SO}}_{4}^{\bullet -}+{\mathrm{SO}}_{4}^{2-}$$

In Cl^–^ containing systems, the reactions of both Cl^•^ (Eq. (12)) and Cl_2_^•–^ (Eq. (13)) contribute to the oxidation of the solute molecules (S). At the same time, both radicals react also with the water molecules (Eqs. (14) and (15)):

12 $$\begin{array}{cc}\mathrm{Cl}^\bullet+\mathrm S\rightarrow\mathrm{organic}\;\mathrm{radical}&k_{\mathrm{Cl}\bullet}\end{array}$$

13 $$\mathrm{Cl}_2^{\bullet-}+\mathrm S\rightarrow2\mathrm{Cl}^-+\mathrm{organic}\;\mathrm{radical}\;\mathrm{cation}$$

14 $$\begin{array}{cc}\mathrm{Cl}^\bullet+{\mathrm H}_2\mathrm O\rightarrow\mathrm{products}&k_{14\;}\left[{\mathrm H}_2\mathrm O\right]=2.5\times10^5\;\mathrm s^{-1}\end{array}$$

15 $$\begin{array}{cc}\mathrm{Cl}^\bullet+{\mathrm H}_2\mathrm O\rightarrow\mathrm{HOClH}+\mathrm{Cl}^-&k_{15\;}\left[{\mathrm H}_2\mathrm O\right]=1300\;\mathrm s^{-1}\end{array}$$

In the relevant works, complex kinetic models were used to derive *k*_Cl•_ (Buxton et al. 1998, 2001; Alegre et al. 2000; Mártire et al. 2001). Mártire et al. in their time resolved experiments under conditions when Reaction (14) can be neglected described the apparent rate constant of Cl_2_^•–^ decay by the equation:

16 $${k}_{\mathrm{app}}= {k}_{15} + \left[\frac{{k}_{\mathrm{Cl}\bullet }}{K\left[{\mathrm{Cl}}^{-} \right]}+ {k}_{13}\right]\left[\mathrm{S}\right]$$

*K* is the equilibrium constant of Reaction (7). Using this technique, first the experimentally determined *k*_app_ values were plotted against the solute concentration ([S]) at several constant chloride ion concentrations and the slope values were determined. Then, these slope values obtained at several [Cl^–^] were plotted as a function of the reciprocal Cl^–^ concentration (1/[Cl^–^]). The slope of the second plot supplied *k*_Cl•_.

In another group of experimental techniques used in practice, Cl^–^ was not present in the reaction system, such as when Cl^•^ formed in the photolysis of chloroacetone or chloramine. The photolysis of chloroacetone yields Cl^•^ through the decay of the singlet excited molecule (Buxton et al. 2000; Wicktor et al. 2003; Lei et al. 2019). The participation of the triplet excited cloroacetone molecules was disclosed based on the absence of dissolved oxygen effect:

17 $${\mathrm{CH}}_{3}{\mathrm{COCH}}_{2}\mathrm{Cl}+\mathrm{hv}\to {\left[{\mathrm{CH}}_{3}{\mathrm{COCH}}_{2}\mathrm{Cl}\right]}^{*}\to {\mathrm{CH}}_{3}{\mathrm{COCH}}_{2}^{\bullet }+{\mathrm{Cl}}^{\bullet }$$

18 $$\begin{array}{cc}{\mathrm{Cl}}^{\bullet }+{\mathrm{CH}}_{3}{\mathrm{COCH}}_{2}\mathrm{Cl}\to \mathrm{Products}& 1.0\pm 0.1\times {10}^{7}{\mathrm{mol}}^{-1}{\mathrm{dm}}^{3}{\mathrm{s}}^{-1}\end{array}$$

Cl^•^ reacts relatively slowly with chloroacetone, *k*_18_ = 1.0 ± 0.1 × 10^7^ mol^–1^ dm^3^ s^–1^. The chloroacetone concentrations are generally around 10^–2^ mol dm^–3^, at this concentration the transient absorption of Cl^•^ (λ_max_ = 320 nm, *ε*_max_ ≈ 4500 mol^–1^ dm^3^ cm^–1^, Buxton et al. 2000) appears immediately after the pulse. The CH_3_COCH_2_^•^ radical has just a small contribution to the absorbance at 320 nm. In case when the transient products do not absorb significantly at the *λ*_max_ of Cl^•^, the decay kinetics at 320 nm can be used to determine the rate constant. For molecules with products having observable absorbance beyond the absorbance range of the chlorine atom, the rate constant could be monitored by observing the absorbance of the products formed (Lei et al. 2019; Ma et al. 2021).

The absorption signal of organic radicals formed in (Eq. (12)) for most of compounds overlaps with the absorption of Cl^•^. Cl^•^-adducts of benzenes, for example, have absorption maxima at 320–360 nm. In these cases, the competition kinetics method can be used as an alternative in transient experiments. Lei et al. (2019) used chloroacetone to produce Cl^•^, SCN^–^ as a competitor (Eqs. (19) and (20)). They recorded the absorbances of (SCN)_2_^•–^ at 472 nm (*ε*_max_ = 7850 mol^–1^ dm^3^ cm^–1^, Buxton and Stuart 1995) without and with various concentrations of the target compounds.

19 $$\begin{array}{cc}{\mathrm{Cl}}^{\bullet }+{\mathrm{SCN}}^{-}\to {\mathrm{Cl}}^{-}+{\mathrm{SCN}}^{\bullet }& {k}_{19}=5.3\times {10}^{9}{\mathrm{mol}}^{-1}{\mathrm{dm}}^{3}{\mathrm{s}}^{-1}\end{array}$$

20 $${\mathrm{SCN}}^{\bullet }+{\mathrm{SCN}}^{-}\to {\left(\mathrm{SCN}\right)}_{2}^{\bullet -}$$

The competition is described by the following expression:

21 $$\frac{{A}_{0}}{A} = \frac{{k}_{\mathrm{Cl}\bullet }\left[\mathrm{S}\right]}{G + {k}_{19}\left[{\mathrm{SCN}}^{-}\right]}$$

22 $$\mathrm{G}={k}_{14}\left[{\mathrm{H}}_{2}\mathrm{O}\right]+{k}_{18}\left[{\mathrm{CH}}_{3}{\mathrm{COCH}}_{2}\mathrm{Cl}\right]$$

where *A*_0_ is the transient absorbance of [(SCN)_2_^•–^] at 472 nm in the absence of S, *A* is the transient absorbance with S present, [S], [SCN^–^], [H_2_O] and [CH_3_COCH_2_Cl] are the concentrations of S, SCN^–^, H_2_O and CH_3_COCH_2_Cl, respectively. At 0.5 and 10 mmol dm^–3^ SCN^–^ and CH_3_COCH_2_Cl concentrations, respectively, *G* was calculated as 3.6 × 10^5^ s^–1^. The absorbance (*A*) of [(SCN)_2_^•–^] decreased with increasing [S], and the second-order rate constant was then determined by plotting *A*_0_/*A* against the [S]/(*G* + *k*_19_[SCN^–^]) ratio.

In several papers, chloramine was used to produce Cl^•^ (Mangalgiri et al. 2019; Sun et al. 2019; Li et al. 2020). In the photolytic process (Eq. (23)), NH_2_^•^ also forms, this radical is assumed to have low reactivity (Patton et al. 2017):

23 $$\begin{array}{cc}{\mathrm{NH}}_2\mathrm{Cl}+\mathrm{hv}\rightarrow\mathrm{NH}_2^\bullet+\mathrm{Cl}^\bullet&\Phi_{254\mathrm{nm}}=0.29\pm0.03\;\mathrm{mole}\;\mathrm{Einstein}^{-1}\end{array}$$

Recently several authors estimated rate constants based on experiments in the UV/chlorine process. In the experiments used for Cl^•^ rate constant determination, the solutions were spiked with hypochlorous acid/hypochlorite ions (HOCl/OCl^−^) before the measurements to produce Cl^•^ in Reactions (3) and (4). In these experiments as UV light source low-pressure mercury lamp, UV-LED or solar radiation was used (Sun et al. 2019; Cai et al. 2020; Xiang et al. 2020; Kong et al. 2020; Li et al. 2020; Liu et al. 2020). The UV/chlorine kinetic system is rather complicated with many individual reactions participating (Jin et al. 2011). There are two main radical species present ^•^OH and Cl^•^. Usually chlorobenzene and/or benzoic acid and nitrobenzene are used to differentiate the reactions of the two reactive radicals. Chlorobenzene and benzoic acid have high rate constants in reaction with both radicals. The reactivity of Cl^•^ with nitrobenzene was suggested to be low (Watts and Linden 2007; Fang et al. 2014; Bulman et al. 2019), while that of with ^•^OH was high. Therefore, it was assumed that, chlorobenzene or benzoic acid reacted with both Cl^•^ and ^•^OH, while nitrobenzene was assumed to react only with ^•^OH (Fang et al. 2014). The rate constants were generally derived using some simulation or fitting procedures. As we will show, this method often gives unrealistic rate constants differing from the values determined by other techniques by more than one order of magnitude. It should be mentioned, that in a recent article Lei et al. (2020) published quite high rate constant (1.01 × 10^10^ mol^–1^ dm^3^ s^–1^) for the Cl^•^ + nitrobenzene reaction.

Gilbert et al. (1988) applied a special rapid-mixing technique combined with ESR detection for rate constant determination. They produced SO_4_^•–^ by Ti(III) activation of persulfate ions. The method allowed also the identification of the radicals formed.

Compilation of the published rate constants collected from the original publications is given in the tables. In selecting the compounds for tabulation, we concentrated on molecules of environmental concern. The pH values and the accuracies are indicated as they were published in the original works. Most measurements were made around room temperature; very few temperature dependence studies were published. In the tables, the temperature differing from room temperature is indicated. The tables show also the p*K*_a_ values of compounds collected from several publications, e.g., Perrin (1965), Babic et al. (2007), Shalaeva et al. (2008). The error bounds in tables represent the σ-level uncertainty. The methods of *k*_Cl•_ determinations are indicated in the tables by abbreviations: PR pulse radiolysis, FP flash photolysis, LFP laser flash photolysis, Comp. competitive method, LFP, C laser flash photolysis combined with the SCN^–^ technique, Compl. and Fit. simulation/modeling/fitting in complex reaction systems often taking into account large numbers of reactions (usually UV/chlorine), Est. estimated based on rate constant of structurally similar compounds, Calc. quantum chemical calculations.

### *Cl*^•^* reaction with inorganic species*

In Table 1, we collected a large number of rate constants of Cl^•^ reactions with inorganic molecules and ions. Most of these reactions are important from the point of view of the UV/chlorine system. We mentioned some of these reactions before in connection with the basic chemistry of Cl^•^ and the rate constant measuring techniques. Most of rate constants of reactions with inorganic species in Table 1 are in the 10^8^–10^9^ mol^–1^ dm^3^ s^–1^ range.

**Table 1:** Rate constant of Cl^•^ reaction with inorganic species

Species	*k*_Cl•_, mol^–1^ dm^3^ s^–^^1^	Method, pH	Reference
H_2_O	1.6×10^5^ s^–^^1^	FP	Klaning and Wolff 1985
	2.5 ± 0.2×10^9^	LFP	McElroy 1990
	2.5 ± 0.5×10^5^ s^–^^1^	LFP	Buxton et al. 1998
	1.6 ± 0.2×10^5^ s^–^^1^	LFP	Yu et al. 2004
Cl^–^	2.1×10^10^	PR	Jayson et al. 1973
	6.5×10^9^	FP	Klaning and Wolff 1985
	8.0×10^9^	FP	Nagarajan and Fessenden 1985
	8.5 ± 0.7×10^9^	LFP	Buxton et al. 1998
	7.8 ± 0.8×10^9^	Averaged	Yu and Barker 2003
HOCl	2.0×10^9^		Zehavi and Rabani 1972
	3.0×10^9^	FP	Klaning and Wolff 1985
ClO^–^	8.2×10^9^	FP	Klaning and Wolff 1985
ClO_3_^–^	5.0 ± 0.1×10^8^	LFP	Buxton et al. 2000
OH^–^	1.8×10^10^	FP	Klaning and Wolff 1985
HSO_3_^–^	2.8 ± 0.3×10^9^	LFP	Buxton et al. 2000
SO_4_^2–^	2.5×10^8^	FP	Huie et al. 1991
	1.7 ± 0.2×10^8^	LFP	Buxton et al. 2000
S_2_O_8_^2–^	8.8 ± 0.5×10^6^	LFP	Yu et al. 2004
CO_3_^2–^	5×10^8^	Comp	Mertens and von Sonntag 1995
HCO_3_^–^	2.2×10^8^	Comp	Mertens and von Sonntag 1995
	2.4 ± 0.5×10^9^	LFP	Buxton et al. 2000
H_2_O_2_	4 × 10.^9^	Est	Matthew and Anastasio 2006
	2.0 ± 0.3×10^9^	LFP	Yu and Barker 2003
Fe^2+^	1.3×10^10^	PR	Bjergbakke et al. 1987
NO_3_^–^	1.0 ± 0.1×10^8^	LFP	Buxton et al. 2000
NO_2_^–^	5.0 ± 0.2×10^9^	LFP	Buxton et al. 2000
OCN^–^	2.2 ± 0.4×10^9^	LFP	Buxton et al. 2000
SCN^–^	5.3 ± 0.3×10^9^	LFP	Buxton et al. 2000
	5.3×10^9^	LFP	Lei et al. 2019
N_3_^–^	5.2 ± 0.4×10^9^	LFP	Buxton et al. 2000
NH_2_Cl	10^8^–10^9^	Est	Wu et al. 2019

The reaction between the chlorine atom and the water molecules (Eq. (14)) is very important from the point of view of the UV/chlorine technique, actually it determines the lifetime of Cl^•^, and strongly influences the kinetic measurements. As Eqs. (24) and (27) show, in two-step equilibrium processes hydroxyl radical and chloride ion is suggested to be produced in the reaction (Klaning and Wolff 1985; McElroy 1990; Buxton et al. 1998; Yu et al. 2004). However, as McElroy (1990) mentions the experimental observations are not entirely consistent with this mechanism, particularly the apparent absence of any dependence on pH.

24 $$\begin{array}{cc}{\mathrm{Cl}}^{\bullet }+{\mathrm{H}}_{2}\mathrm{O}\rightleftharpoons {\mathrm{ClOH}}^{\bullet }+{\mathrm{H}}^{+}& {k}_{24}\left[{\mathrm{H}}_{2}\mathrm{O}\right]=2.5\times {10}^{5}{\mathrm{s}}^{-1}\end{array}$$

-24 $${k}_{-24}=2.6\times {0.6\times 10}^{10}{\mathrm{mol}}^{-1}{\mathrm{dm}}^{3}{\mathrm{s}}^{-1}$$

25 $$\begin{array}{cc}{\mathrm{ClOH}}^{\bullet -}\rightleftharpoons {}^{\bullet }\mathrm{OH}+{\mathrm{Cl}}^{-}& {k}_{25}=6.1\pm 0.8\times {10}^{9}{\mathrm{s}}^{-1}\end{array}$$

-25 $${k}_{-25}=4.3\times {0.4\times 10}^{9}{\mathrm{mol}}^{-1}{\mathrm{dm}}^{3}{\mathrm{s}}^{-1}$$

The rate constant of Cl^•^ reaction with chloride ion is very high (Eq. (7), 7.8 ± 0.8 × 10^9^ mol^–1^ dm^3^ s^–1^, Yu and Barker 2003), the first step of the dimer radical anion (Cl_2_^•–^) formation is followed by several equilibrium reactions, at the end hydroxyl radical can form. At higher pH (above 5) ^•^OH formation is favored. These reactions were detailed in a publication of Buxton et al. (1998), but in our previous review paper on the reactions of Cl_2_^•–^ with organic molecules of environmental interest we also summarized the mechanism (Wojnárovits and Takács 2021).

In the reactions of Cl^•^ with HOCl and ClO^–^ chlorine monoxide radical (ClO^•^, *E*^0^(ClO^•^/ClO^–^ = 1.39 V *vs*. NHE, Armstrong et al. 2015) forms, this radical is a much milder oxidant as Cl^•^. Therefore, these reactions decrease the oxidizing capacity during the UV/chlorine process (Zehavi and Rabani 1972; Klaning and Wolff 1985). The high rate constant of the Cl^•^ + OH^–^ reaction (1.8 × 10^10^ mol^–1^ dm^3^ s^–1^, Klaning and Wolff 1985), in which similarly to the reaction with H_2_O ClOH^•–^ forms, restricts the possibility for the investigations of Cl^•^ reactions to the lower pH region.

A possible combination of UV/chlorine and the Fenton technique is strongly influenced by the high rate constants of the Cl^•^ + H_2_O_2_ and Cl^•^ + Fe^2+^ reactions: 2.0 ± 0.3 × 10^9^ and 1.3 × 10^10^ mol^–1^ dm^3^ s^–1^, respectively (Yu and Barker 2003; Bjergbakke et al. 1987). The water to be treated always contains some bicarbonate/carbonate ions. Their reactions with Cl^•^ give carbonate radical anions (CO_3_^•–^, *E*^0^(CO_3_^•–^/CO_3_^2–^ = 1.57 V and *vs*. NHE, Armstrong et al. 2015), a radical anion with lower oxidizing ability and higher selectivity as Cl^•^ (Mertens and von Sonntag 1995).

The rate constants of reactions with most ions (possible impurities in water) take place by single-electron-transfer (SET) mechanism (Buxton et al. 2000).

### Simple oxidized molecules

**Scheme 1.**
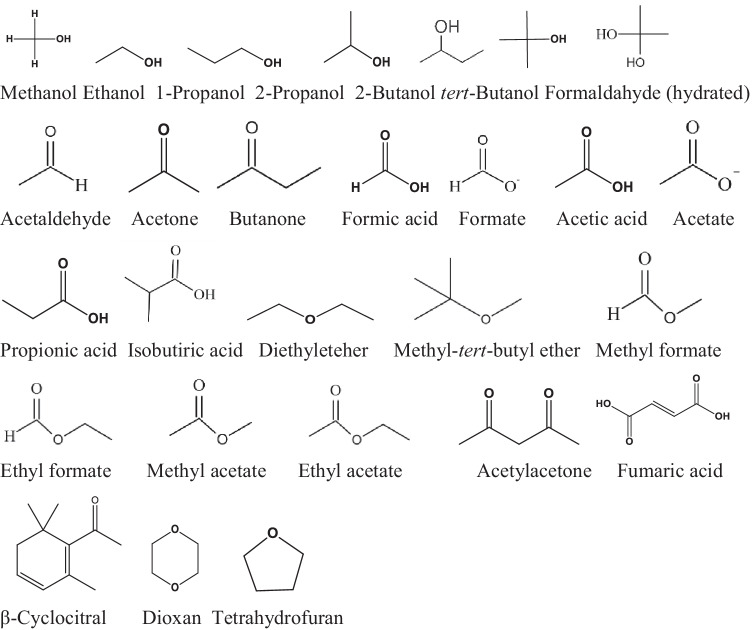
Simple oxidized molecules

A large number of rate constants are available on Cl^•^ reactions with simple oxidized molecules (Scheme 1) (Gilbert et al. 1988; Mertens and von Sonntag 1995; Buxton et al. 2000; Wicktor et al. 2003): all values are in the 10^8^–10^9^ mol^–1^ dm^3^ s^–1^ range (Table 2). In the experiments of Gilbert et al. (1988), Cl^•^ was generated in the reaction of C1^–^ with SO_4_^•–^ and H_2_PO_4_^•–^ obtained by metal-catalyzed decomposition of the appropriate peroxides. Buxton et al. (2000) and Wicktor et al. (2003) used the laser flash photolysis (LFP) technique for Cl^•^ production in chloroacetone photodecomposition and they calculated the rate constants using the decay of Cl^•^ absorbance. Mertens and von Sonntag (1995) determined the *k*_Cl•_ values in competitive reactions. Minakata et al. (2017) conducted detailed quantum mechanical calculations on the mechanisms. In the case of several molecules, e.g., methanol, ethanol, similar rate constants were measured in two or three laboratories. Generally, the values agreed with each other within one order of magnitude. The authors suggested H-abstraction and Cl-adduct formation as the main mechanisms.

**Table 2:** Simple oxidized molecules

Compound, p*K*_a_	*k*_Cl•_, mol^–1^ dm^3^ s^–^^1^	Method, pH	Reference
Methanol	1.0 ± 0.2×10^9^	LFP, 6	Buxton et al. 2000
	1.0 ± 0.1×10^9^	LFP, 5.4	Wicktor et al. 2003
	9.0×10^9^	LFP, 7	Lei et al. 2019
Ethanol	2.25×10^9^	Compl., ESR, 2	Gilbert et al. 1988
	1.7 ± 0.3×10^9^	LFP, 6	Buxton et al. 2000
	2.2 ± 0.3×10^9^	LFP, 5.4	Wicktor et al. 2003
1-Propanol	2.2 ± 0.4×10^9^	LFP, 5.4	Wicktor et al. 2003
2-Propanol	6×10^9^	PR	Mertens and von Sonntag 1995
	1.5 ± 0.2×10^9^	LFP, 6	Buxton et al. 2000
	3.2 ± 0.7×10^9^	LFP, 5.4	Wicktor et al. 2003
2-Butanol	5.0 ± 0.6×10^9^	LFP, 5.4	Wicktor et al. 2003
*tert*-Butanol	2.2×10^9^	Compl., ESR, 2	Gilbert et al. 1988
	3×10^8^	PR	Mertens and von Sonntag 1995
	3.2×10^8^	LFP, 6, 5 °C	Buxton et al. 2000
	4.78×10^8^	LFP, 6, 15 °C	
	6.2 ± 0.3×10^8^	LFP, 6, 25 °C	
	8.51×10^8^	LFP, 6, 35 °C	
	1.5 ± 0.1×10^9^	LFP, 5.4	Wicktor et al. 2003
Formaldehyde (hydrated)	1.4 ± 0.1×10^9^	PR, 6	Buxton et al. 2000
	1.4 ± 0.3×10^9^	LFP, 5.4	Wicktor et al. 2003
Acetaldehyde	6.3 ± 0.4×10^8^	LFP, 6	Buxton et al. 2000
Acetone	< 5×10^6^	LFP, 6	Buxton et al. 2000
	7.8 ± 0.7×10^7^	LFP, 5.4	Wicktor et al. 2003
2-Butanone	2.4 ± 0.3×10^8^	LFP, 5.4	Wicktor et al. 2003
Formic acid, 3.75	1.3 ± 0.1×10^8^	LFP, 1	Buxton et al. 2000
	2.8 ± 0.3×10^9^	LFP, 1	Wicktor et al. 2003
Formate	4.2 ± 0.1×10^9^	LFP, 6	Buxton et al. 2000
Acetic acid, 4.75	2×10^8^	Compl., ESR, 2	Gilbert et al. 1988
	3.2 ± 0.2×10^7^	LFP, 1	Buxton et al. 2000
	1.0 ± 0.2×10^8^	LFP, 1	Wicktor et al. 2003
Acetate	3.7 ± 0.4×10^9^	PR, 6	Buxton et al. 2000
Propionic acid, 4.88	8×10^8^	Compl., ESR, 2	Gilbert et al. 1988
	1.2 ± 0.3×10^9^	LFP, 1	Wicktor et al. 2003
Isobutyric acid, 4.86	1.7 ± 0.3×10^9^	LFP, 1	Wicktor et al. 2003
Diethyl ether	1.3 ± 0.1×10^9^	LFP, 5.8	Wicktor et al. 2003
Methyl-tert-butyl ether	1.3 ± 0.1×10^9^	LFP, 5.4	Wicktor et al. 2003
Methyl formate	2.0 ± 0.1×10^7^	LFP, 4	Buxton et al. 2001
Ethyl formate	7.2 ± 0.2×10^7^	LFP, 4	Buxton et al. 2001
Methyl acetate	1.4 ± 0.1×10^7^	LFP, 4	Buxton et al. 2001
Ethyl acetate	8.0 ± 0.7×10^7^	LFP, 4	Buxton et al. 2001
Acetylacetone, 8.9	2.9 ± 0.3×10^9^	Comp., 7	Lei et al. 2019
Fumaric acid, 3.03, 4.44	3~3×10^9^	Compl., ESR, 2	Gilbert et al. 1988
β-Cyclocitral	9.58 ± 0.38 × 10^9^	Comp, 7	Xiang et al. 2020
1,4-Dioxan	2.8–3.4×10^9^	Est	Li et al. 2017
	4.38 ± 0.38×10^6^	PR, 5.8	Patton et al. 2017
Tetrahydrofuran	2.6 ± 0.4×10^9^	LFP, 5.4	Wicktor et al. 2003

### Alcohols

Buxton et al. (2000) and Wicktor et al. (2003) published *k*_Cl•_=1 × 10^9^ mol^–1^ dm^3^ s^–1^ for methanol (Scheme 1). For alcohols with a higher C-atom number, the values seem to be somewhat bigger. The average of the *k*_Cl•_ values published by Gilbert et al. (1988), Buxton et al. (2000) and Wicktor et al. (2003) for the reaction of ethanol is 2 × 10^9^ mol^–1^ dm^3^ s^–1^. The values published by Mertens and von Sonntag (1995), Buxton et al. (2000) and Wicktor et al. (2003) for 2-propanol are highly different, they are 6 × 10^9^, 1.5 ± 0.1 × 10^9^ and 3.2 ± 0.7 × 10^9^ mol^–1^ dm^3^ s^–1^, respectively. The same is true for *tert*-butanol: the values of Mertens and von Sonntag (1995) and Buxton et al. (2000) are 3 × 10^8^ and 6.2 ± 0.3 × 10^8^ mol^–1^ dm^3^ s^–1^, respectively, whereas the values of Gilbert et al. (1988) and Wicktor et al. (2003) are 2.2 ± 0.3 × 10^9^ and 1.1 ± 0.1 × 10^8^ mol^–1^ dm^3^ s^–1^, respectively. 1-Propanol and 2-butanol react with *k*_Cl•_ values of 2.2 ± 0.4 × 10^9^ and 5.0 ± 0.6× 10^9^ mol^–1^ dm^3^ s^–1^, respectively (Wicktor et al. 2003).

The H-abstraction from ethanol may take place from both the α and β carbon atom; Gilbert et al. (1988) suggest a 2:1 ratio of the two reactions (bond-strength effect). In the case of *tert*-butanol, Cl^•^ reacts with the H-atoms on the methyl groups and with the alcoholic OH in 2:1 ratio. The abstraction reaction from OH proceeds with an electron transfer mechanism (a process in which the aqueous environment would be expected to stabilize the incipient charges). The reaction is suggested to proceed in the following way:

26 $${\left({\mathrm{CH}}_3\right)}_3\mathrm{COH}+\mathrm{Cl}^\bullet\rightarrow\left[\mathrm{Cl}^-+\left({\mathrm{CH}}_3\right)_3^{\bullet+}\mathrm{COH}\right]\xrightarrow{{-\mathrm{Cl}}^-,-\mathrm H^+}{\left({\mathrm{CH}}_3\right)}_3\mathrm{Cl}^\bullet\rightarrow{\left({\mathrm{CH}}_3\right)}_2\mathrm{CO}+{\mathrm{CH}^\bullet}_3$$

The methyl radicals observed support the mechanism.

### Aldehydes and ketones

In aqueous solutions aldehydes and ketones undergo hydration, the extent of which strongly depends on the chemical structure. We show this hydration on the example of a ketone:27

The hydration does not affect the number of available C-H bonds, but it does introduce O–H groups to the molecule with which Cl^•^ is known to react (Buxton et al. 2000). There may be a relationship between the H^•^ atom abstraction rate constant and the extent of hydration of the carbonyl species. HCHO and CH_3_CHO are hydrated to ~99 and ~45% extent, respectively, which is reflected also by the significant decrease of the rate constants between formaldehyde and acetaldehyde, 1.4 ± 0.3 × 10^9^ and 6.3 ± 0.4×10^8^ mol^–1^ dm^3^ s^–1^, respectively (Buxton et al. 2000; Wicktor et al. 2003).

The rate constant of 2-butanone is much smaller, 2.4 ± 0.3×10^8^ mol^–1^ dm^3^ s^–1^ (Wicktor et al. 2003), than those measured for formaldehyde and acetaldehyde. For acetone highly different values were published by Buxton et al. (2000) and Wicktor et al. (2003): < 5×10^6^ and 7.8 ± 0.7×10^7^ mol^–1^ dm^3^ s^–1^, respectively.

### Acids, acid esters

Buxton et al. (2000) published much smaller rate constant (1.3 ± 0.1 × 10^8^ mol^–1^ dm^3^ s^–1^) for the formic acid + Cl^•^ reaction as determined by Wicktor et al. (2003) (2.8 ± 0.3×10^9^ mol^–1^ dm^3^ s^–1^). The latter authors published high values also for the reactions of propionic and isobutyric acids: 1.2 ± 0.3×10^9^ and 1.7 ± 0.3×10^9^ mol^–1^ dm^3^ s^–1^, respectively. The values published for acetic acid in three laboratories are c.a. one order of magnitude smaller than those of the previously mentioned compounds (Gilbert et al. 1988; Buxton et al. 2001; Wicktor et al. 2003). Otherwise, the dominant reaction mechanism for carboxylic acids and carboxylates, except formic acid and formate, is H-abstraction from a C-H bond. Formate and formic acid undergo Cl-adduct formation predominantly (Minakata et al. 2017).

### Simple chloro- and sulfo-compounds

**Scheme 2.**
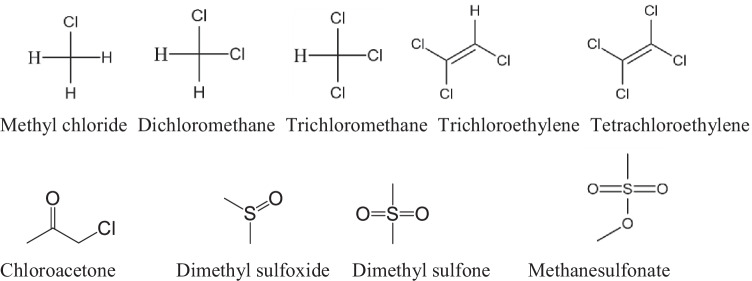
Simple chloro- and sulfo compounds

Cl^•^ reacts with chlorinated methanes and chloroacetone (Scheme 2) by H-abstraction reaction (Buxton et al. 1999, 2000, 2001; Wicktor et al. 2003). The rate constant values (10^7^–10^8^ mol^–1^ dm^3^ s^–1^, Table 3) are determined by the C-H bond energy of the attacked bond as in the case of simple oxygenated molecules.

**Table 3:** Simple chloro- and sulfo-compounds

Compound	*k*_Cl•_, mol^–1^ dm^3^ s^–^^1^	Method, pH	Reference
Methyl chloride	2.3 ± 0.5×10^8^	LFP, 5.4	Buxton et al. 2001
Dichloromethane	4.7 ± 0.3×10^6^	LFP, 5.4	Buxton et al. 2001
	9.3 ± 0.3×10^6^	LFP, 5.4	Wicktor et al. 2003
Trichloromethane	2.3 ± 0.5×10^8^	LFP, 5.4	Wicktor et al. 2003
Trichloroethylene	1.9×10^8^	Compl	Li et al. 2007
Tetrachloroethylene	2.8×10^8^	Comp	Mertens and von Sonntag 1995
Chloroacetone	9.7 ± 1.5×10^6^	PR	Buxton et al. 1999
	1.1 ± 0.1×10^7^	PR, 6	Buxton et al. 2000
	1.3 ± 0.2×10^7^	PR, 6	
Dimethyl sulfoxide	6.3 ± 0.6 × 10^9^	LFP, 5–6	Zhu et al. 2005
Dimethyl sulfone	8.2 ± 1.6×10^5^	LFP, 5–6	Zhu et al. 2005
Methanesulfonate	4.9 ± 0.2×10^5^	LFP, 5–6	Zhu et al. 2005

The reactions with the unsaturated compounds, trichloroethylene and tetrachloroethylene take place by Cl^•^ addition to the double bond (Mertens and von Sonntag 1995; Li et al. 2007). The chlorine atom reactions with substituted olefins occur with rate constants values (1.9×10^8^ and 2.8×10^8^ mol^–1^ dm^3^ s^–1^, respectively) smaller by approximately one order of magnitude than those for the OH radical reactions. In the presence of dissolved O_2_, the Cl^•^ addition reaction is followed by peroxyl radical formation and a short chain reaction starts in which Cl^•^ is the chain carrier released in the bimolecular termination reactions of the various peroxyl radicals formed in the system (Mertens and von Sonntag 1995). In these reactions, highly poisonous phosgene (COCl_2_) may also form.

**Scheme 3.**
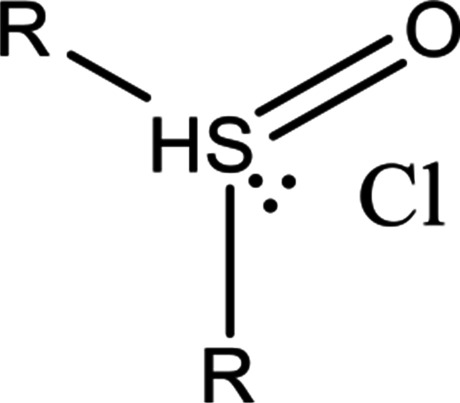
Three-electron bonded complex formed in alkyl sulfoxide + Cl^•^ reaction

Oxidation of dialkyl sulfides in air droplets may play an important role in modifying the global climate since several of their free radical induced oxidation products are water soluble contributing to atmospheric aerosols formation (Zhu et al. 2005). Dimethyl sulfoxide, the slightly oxidized form of dimethyl sulfide reacts with relatively high rate constant of 6.3 ± 0.6×10^9^ mol^–1^ dm^3^ s^–1^, while the reactivities of the more oxidized forms, dimethyl sulfone and methanesulfonate are much smaller, 8.2 ± 1.6 × 10^5^ and 4.9 ± 0.2×10^5^ mol^–1^ dm^3^ s^–1^, respectively. This is a general trend observed in one electron oxidation of sulfur containing molecules: the reactivities of molecules with S-atom in low oxidized state are much higher than those of molecules with high-oxidized S-atom. Zhu et al. (2005) carried out these measurements in laser photolysis experiments in systems where both Cl^•^ and Cl_2_^•–^ reactive intermediates were present. Transient kinetic spectroscopic measurements in aqueous solutions with dimethyl sulfoxide revealed the formation of chlorine atom–sulfur three electron bonded complexes (Scheme 3). These complexes are generally observed intermediates in one-electron oxidation of sulfur compounds. They are characterized by a sulfur-chlorine three-electron bond with two σ-bonding and one σ*-antibonding electron (Asmus 1990). In a system, where both Cl^•^ and Cl_2_^•–^ participate in the oxidation the complexes may form in several reactions. However, in carbon tetrachloride solution (no Cl_2_^•–^) Sumiyoshi and Katayama (1987) suggested Cl^•^ addition to the S-atom of dimethyl sulfoxide based on spectral and kinetic studies, *k*_Cl•_ = 7.5 ± 0.2×10^9^ mol^–1^ dm^3^ s^–1^.

### Simple aromatic molecules

**Scheme 4.**
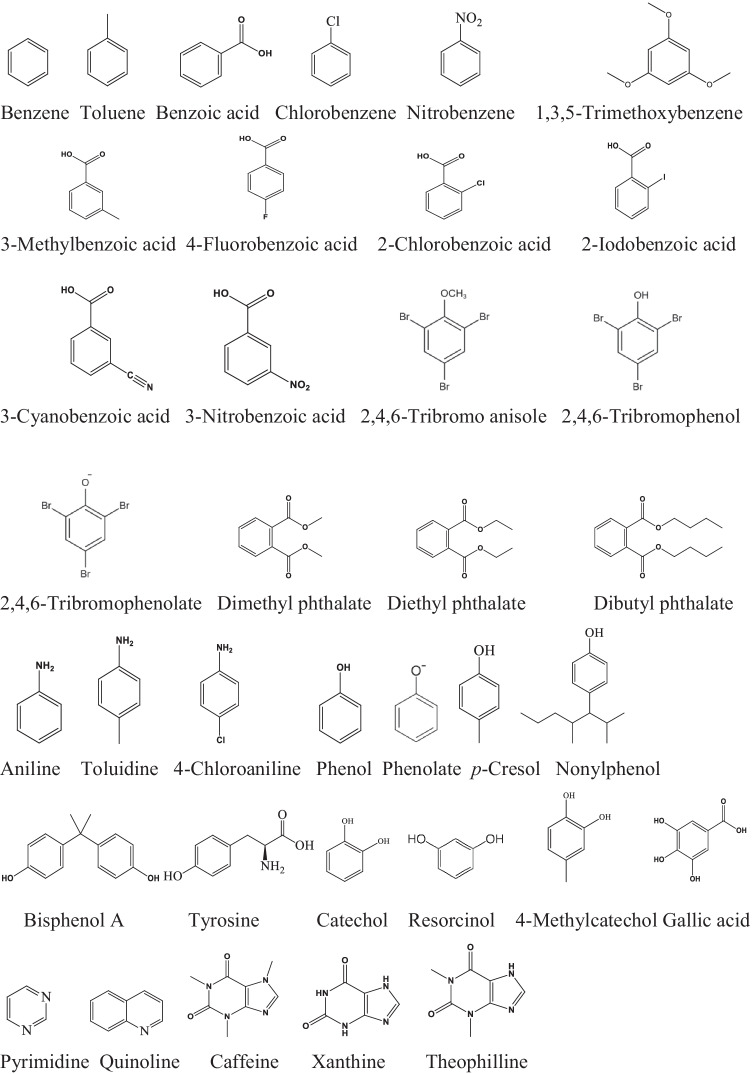
Aromatic molecules

Most of the pharmaceutical, pesticide, personal care, etc., compounds contain benzene ring in their structures. Here, in this chapter we discuss the reactions of Cl^•^ with relatively simple aromatic molecules (Scheme 4). Mártire et al. (2001) reported the same *k*_•Cl_ values for toluene, chlorobenzene and benzoic acid: 1.8 ± 0.3×10^10^ mol^–1^ dm^3^ s^–1^ (Table 4). In their former publication, these authors for benzene gave a value of 6.0×10^9^ ≤ *k*_Cl•_ ≤ 1.2×10^10^ mol^–1^ dm^3^ s^–1^ (Alegre et al. 2000). Watts and Linden (2007) analyzing the reactions in complex aqueous systems containing organic molecules assumed that nitrobenzene reacts with negligible rate with Cl^•^, at the same time it is highly reactive toward ^•^OH. According to the recent experiments of Lei et al. (2021), the rate constant of the Cl^•^ + nitrobenzene reaction is not negligible, but rather high, 1.01×10^10^ mol^–1^ dm^3^ s^–1^.

**Table 4:** Simple aromatic molecules

Compound, p*K*_a_	*k*_Cl•_, mol^–1^ dm^3^ s^–^^1^	Method, pH	Reference
Benzene	6.0×10^9^–1.2×10^10^	LFP, 2.5–3	Alegre et al. 2000
Toluene	1.8 ± 0.3×10^10^	LFP, 3–4	Mártire et al. 2001
Benzoic acid, 4.2	1.8 ± 0.3×10^10^	LFP, 3–4	Mártire et al. 2001
	1.35 ± 0.15×10^10^	LFP,C, 7	Lei et al. 2019
Chlorobenzene	1.8 ± 0.3×10^10^	LFP, 3–4	Mártire et al. 2001
Nitrobenzene	Negligible	Est	Watts and Linden 2007
	Negligible	Est	Bulman et al. 2019
	1.01×10^10^	LFP,C, 7	Lei et al. 2021
TMB (1,3,5-Trimethoxybenzene)	1.33 ± 0.08×10^10^	LFP,C, 3	Lei et al. 2019
	8.3 ± 0.18×10^9^	LFP, 3	
3-Methylbenzoate, 4.27	1.64×10^9^	Est., 7.2	Zhou et al. 2019
4-Fluorobenzoate, 4.14	7.92×10^8^	Est., 7.2	Zhou et al. 2019
2-Chlorobenzoate, 2.9	6.00×10^8^	Est, 7.2	Zhou et al. 2019
2-Iodobenzoate, 2.86	3.85×10^8^	Est., 7.2	Zhou et al. 2019
3-Cyanobenzoate, 3.6	6.35×10^7^	Est., 7.2	Zhou et al. 2019
3-Nitrobenzoate, 3.4	4.18×10^7^	Est., 7.2	Zhou et al. 2019
2,4,6-Tribromoanisole	7.14×10^10^	Calc	Li et al. 2021
2,4,6-Tribromophenol	5.54×10^10^	Calc	Li et al. 2021
2,4,6-Tribromophenolate	≥ 2.36×10^10^	Calc	Li et al. 2021
Dimethyl phthalate	1.81 ± 0.18×10^10^	LFP,C, 7	Lei et al. 2019
	1.8×10^10^	LFP,C, 7	Lei et al. 2020
Diethyl phthalate	1.97 ± 0.14×10^10^	LFP,C, 7	Lei et al. 2019
	2.0×10^10^	LFP,C, 7	Lei et al. 2020
Dibutyl phthalate	1.96 ± 0.22×10^10^	LFP,C., 7	Lei et al. 2019
	2.0×10^10^	LFP,C, 7	Lei et al. 2020
Aniline, 4.63	4.0×10^10^	Est	Li 2017
	2.74 ± 0.31×10^10^	LFP,C, 7	Lei et al. 2019
*p*-Toluidine	2.73 ± 0.56×10^10^	LFP,C, 7	Lei et al. 2019
4-Chloroaniline, 4.15	2.17 ± 0.14×10^10^	LFP,C, 7	Lei et al. 2019
Phenol, 10.0	1.4×10^9^	Est	Grebel et al. 2010
	1.12 ± 0.09×10^10^	LFP,C, 7	Lei et al. 2019
	9.0 ± 1.2×10^9^	LFP, 7	
Phenolate	9.6×10^9^	Est	Grebel et al. 2010
*p*-Cresol, 10.3	1.81×10^10^	Comp	Shruti Salil 2018
Nonylphenol	1.00 ± 0.07×10^10^	LFP,C, 7	Lei et al. 2019
Bisphenol A	1.82 ± 0.23×10^10^	LFP,C, 7	Lei et al. 2019
	1.45 ± 0.08×10^10^	LFP, 7	
Tyrosine, 2.2, 9.1	1.15×10^10^	LFP,C, 7	Lei et al. 2021
Catechol	2.82 ± 0.33×10^10^	LFP,C, 7	Lei et al. 2019
Resorcinol	1.4×10^10^	LFP,C, 7.5	Zhang et al. 2022
4-Methylcatechol	2.49 ± 0.14×10^10^	LFP,C, 7	Lei et al. 2019
Gallic acid, 4.5, 10.0	1.83 ± 0.27×10^10^	LFP,C, 7	Lei et al. 2019
Pyrimidine	5 ± 1×10^8^	LFP,C, 7	Lei et al. 2019
Quinoline	1.2×10^10^	LFP	Khanna et al. 1992
Caffeine	1.46×10^10^	Compl., 7	Sun et al. 2016
	3.87 ± 0.35×10^10^	LFP,C, 7	Lei et al. 2019
Xanthine	3.81 ± 0.40×10^10^	LFP,C, 7	Lei et al. 2019
Theophylline	3.98 ± 0.42×10^10^	LFP,C, 7	Lei et al. 2019

Three mechanisms are considered in Cl^•^ reactions with these simple aromatic molecules: single-electron-transfer (SET) from the ring to Cl^•^, H-abstraction from the aromatic ring or from the alkyl side chain, and radical addition to the aromatic ring. The SET reaction pathway was disregarded in the cases of benzene, toluene and benzoic acid, since the expected final products in this mechanism, phenol derivatives were not observed (Mártire et al. 2001). Based on the theoretical calculations of Minakata et al. (2017), H-abstraction from the benzene ring is a minor reaction. In the reaction of toluene, the transient absorption spectrum shows sharp bands around 300 nm characteristic to the benzyl type radical. However, as Mártire et al. (2001) mention benzyl type radical may not necessarily form in H-abstraction from the methyl group, but it can also be produced by HCl elimination from an intermediate radical. In the ^•^OH + toluene system, only 6% of ^•^OH produces benzyl radical (Sehested et al. 1975).

Absorption spectra of the transients in laser and conventional flash photolysis experiments (Alegre et al. 2000; Mártire et al. 2001) reveal that Cl^•^ addition to the aromatic rings forming chlorocyclohexadienyl radicals is the predominant mechanism in agreement with the results of theoretical calculations. ^•^OH reacts with aromatic molecules also in radical addition (Homlok et al. 2020). The reaction may occur in one or two steps. In the two-step process, first a π-complex forms: the radical is not stabilized to a single bond, in the second step the radical may stabilize at one of the double bonds giving the σ-complex. In the one-step process, the σ-complex forms directly. In the experiments of Alegre et al. (2000) and Mártire et al. (2001), with the applied time resolution (50 ns) no π-complex was observed. The addition (localization) may take place to any of the carbon atoms in the ring. Some of these radicals are expected to transform to chlorinated stable products in disproportionation reaction. In air saturated solution of benzene chlorobenzene represented less than 10% of the consumed benzene (Alegre et al. 2000).

In the reactions of substituted benzenes (e.g., toluene, benzoic acid), some selectivity in the sites of radical addition is expected, similarly to the reactions of ^•^OH (Homlok et al. 2020). Due to the lack of stereochemical identification of the individual products, the preferred reaction sites are not reported in the relevant works, the authors simply assume, that the observed transient absorption spectra belong to mixtures of the various chlorocyclohexadienyl type radicals. In the reaction of benzoic acid, chlorobenzoic acid isomers and also chlorobenzene were identified as final products. The latter involves a mechanism with COOH→Cl exchange.

In the reaction of chlorobenzene, chlorinated phenols were detected (Mártire et al. 2001): they may form through electron transfer from chlorobenzene to Cl^•^ (SET mechanism). The chlorobenzene cation is suggested to undergo a rapid hydration to hydroxycyclohexadienyl radical. This radical in the subsequent reaction may transform to chlorinated phenols. SET mechanism was proven by Lei et al. (2019) in the Cl^•^ reaction of several substituted aromatic molecules, e.g., 1,3,5-trimethoxybenzene (*k*_Cl•_ ≈ 1.0×10^10^ mol^–1^ dm^3^ s^–1^). Under acidic conditions, these radical cations were shown to be long-lived enough to be detected by the usual transient kinetics techniques. At neutral and alkaline pH, they undergo very fast decomposition.

In a recent paper, Zhou et al. (2019) estimated the rate constants of reactions of several reactive species (^•^OH, Cl^•^, Cl_2_^•–^ and ClO^•^) participating in the UV/chlorine process with a series of ionized benzoic acid derivatives at pH 7.2 (3-methyl-, 4-fluoro-, 2-chloro-, 2-iodo- and 3-nitrobenzoate). They set up a kinetic model considering enormously large number of chemical reactions and used fitting procedure to obtain the rate constants. The logarithms of rate constants for all radicals showed good correlations with the Hammett substituent constants with slope values -0.54 (^•^OH), -2.13 (Cl^•^), -0.96 (Cl_2_^•–^) and -0.45 (ClO^•^). Based on the slope values, Cl^•^ seems to be a highly selective one-electron oxidant, more selective than, e.g., Cl_2_^•–^. However, this suggestion is in disagreement with the general observations (e.g., Lei et al. 2019), in which Cl^•^ seems to be less selective. The *k*_Cl_^•^ values of Zhou et al. (2019) for the substituted benzoic acids are 1–3 orders of magnitude smaller than the rate constant measured for benzoic acid at similar pH (1.35 ± 0.15×10^10^ mol^–1^ dm^3^ s^–1^, Lei et al. 2019).

Just the opposite is the case with the rate constant calculated by Li et al. (2021) for 2,4,6-tribromoanisole, 2,4,6-tribromophenol and 2,4,6-tribromophenolate, 7.14×10^10^, 5.54×10^10^ and ≥ 2.36×10^10^ mol^–1^ dm^3^ s^–1^, respectively. The values for the first two are unrealistically high. Moreover, the ionization increases the electron density on the ring. Therefore, higher rate constant is expected for 2,4,6-tribromophenolate than for 2,4,6-tribromophenol.

The rate constants of Cl^•^ reactions with dimethyl-, diethyl and tributyl phthalates (1.8×10^10^–2.0×10^10^ mol^–1^ dm^3^ s^–1^, Lei et al. 2019, 2020) agree with the value published for the neutral benzoic acid (1.8×10^10^ mol^–1^ dm^3^ s^–1^, Mártire et al. 2001). The radical attack is assumed to occur on the aromatic ring.

The rate constants of simple anilines and phenols in Table 4 (aniline, *p*-toluidine, 4-chloroaniline, phenol, *p*-cresol, catechol, resorcinol and 4-methylcatechol) are also very high; they are in the 1×10^10^–4×10^10^ mol^–1^ dm^3^ s^–1^ range (Li 2017; Lei et al. 2019; Zhang et al. 2022). All values are around the diffusion-controlled limit; this can be the reason that the typical rate enhancing/decreasing effect of the substituents is not observed in the *k*_Cl•_ values. For instance, in spite of the fact that chloroaniline has an electron withdrawing substituent, while *p*-toluidine has an electron releasing substituent on the ring the rate constants of both molecules are similar to that of aniline.

Molecules with N atom in the aromatic ring generally have low rate constants in radical reactions (Wojnárovits and Takács 2021). That is true also for the Cl^•^ + pyrimidine reaction, which has a *k*_Cl•_ value of 5 ± 1×10^8^ mol^–1^ dm^3^ s^–1^ (Lei et al. 2019). At the same time, the published rate constant values for quinoline (1.2×10^10^ mol^–1^ dm^3^ s^–1^) caffeine, xanthine and theophylline (~3×10^10^ mol^–1^ dm^3^ s^–1^) are high.

### Pesticides

DEET (*N*,*N*-diethyl-*m*-toluamide) is an often applied insect repellent, it is poorly soluble in water. The published rate constant of reaction with Cl^•^ 3.8×10^9^ mol^−1^ dm^3^ s^−1^ (Sun et al. 2016), was obtained in competitive experiments. It is much smaller than the values published for aniline derivatives. Mecoprop (methylchlorophenoxypropionic acid) is a commonly used herbicide, *k*_Cl•_ = 1.08 × 10^10^ mol^–1^ dm^3^ s^–1^ (Kong et al. 2020) was reported as measured also in competitive experiments.

**Scheme 5.**
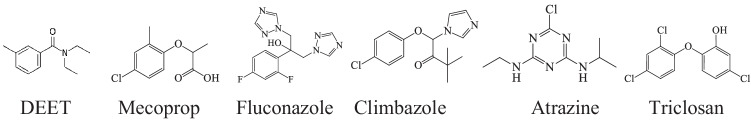
Pesticides

Fluconazole and climbazole are used as fungicides (Scheme 5, Table 5). In fluconazole the two electron withdrawing F-atoms decrease the reactivity with the aromatic ring, and the published rate constant, 5.5×10^9^ mol^−1^ dm^3^ s^−1^ (Cai et al. 2020) is smaller than the values of Mártire et al. (2001) published for simple aromatic molecules. The value for climbazole (6.3±1.5×10^10^ mol^−1^ dm^3^ s^−1^, Cai et al. 2019) is unrealistically high. The rate constant was determined in the UV/free chlorine system, in which both Cl^•^ and ^•^OH were reacting radicals and nitrobenzene was used as scavenger of OH. Atrazine reacts with a much smaller rate constant of 6.87×10^9^ mol^–1^ dm^3^ s^–1^ (Kong et al. 2020). Cl^•^ is expected to abstract H-atom from the alkyl side chains.

**Table 5:** Pesticides

Compound, p*K*_a_	*k*_Cl•_, mol^–1^ dm^3^ s^–^^1^	Method, pH	Reference
DEET	3.8×10^9^	Comp., 7	Sun et al. 2016
Mecoprop	1.08×10^10^	Comp., 7.0	Kong et al. 2020
Fluconazole	5.5×10^9^	Comp.,7	Cai et al. 2020
Climbazole	6.3 ± 1.5×10^10^	Comp., 7.0	Cai et al. 2019
Atrazine	6.87×10^9^	Comp., 7.0	Kong et al. 2020
Triclosan, 7.8–8.14	2.76 ± 0.44×10^10^	LFP,C, 7	Lei et al. 2019

The structure of triclosan (fungicide) shows some similarity to that of clofibric acid (discussed later), the high reactivity of triclosan, *k*_Cl•_ = 2.76 ± 0.44×10^10^ mol^−1^ dm^3^ s^−1^, is probably due to the two aromatic rings in the molecule (Lei et al. 2019).

### Antibiotics

**Scheme 6.**
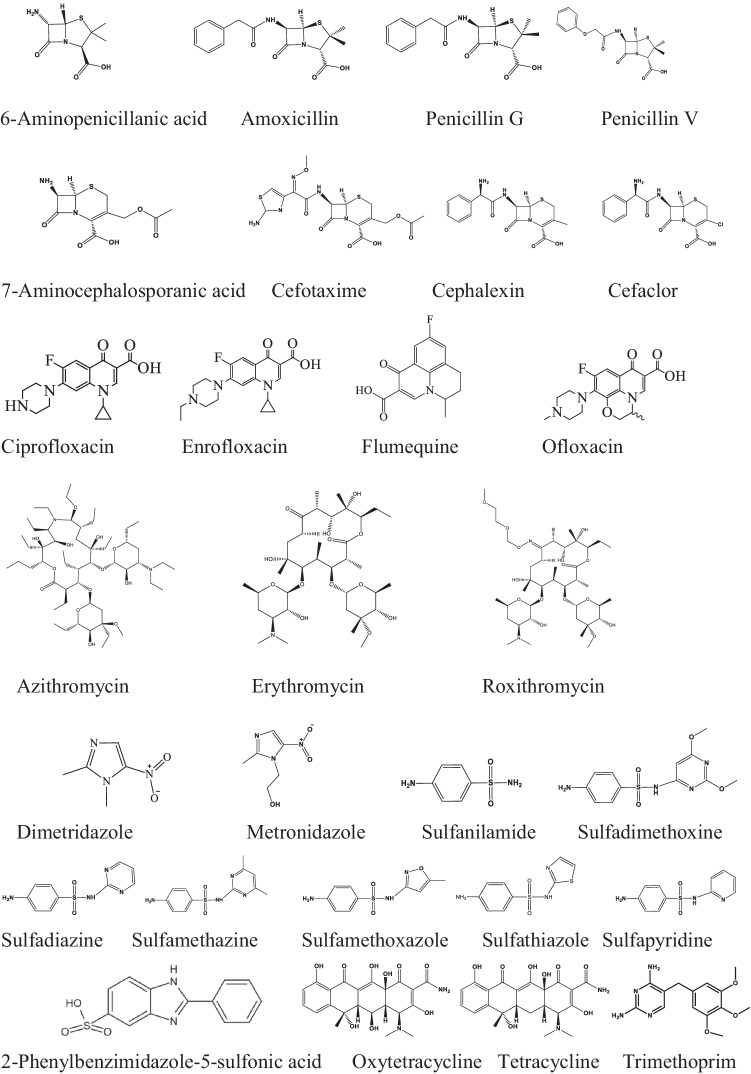
Antibiotics

The rate constant of amoxicillin, penicillin G and penicillin V, *k*_Cl•_ ≈ 1.2×10^10^, is much higher, than measured for their structural unit, 6-aminopenicillanic acid, 3.4 ± 0.3×10^9^ mol^–1^ dm^3^ s^–1^, which is responsible for the antibacterial effect (Scheme 6, Table 6) (Lei et al. 2019). The higher value shows, that not the β-lactam part is the main target of Cl^•^ attack. We assume that the main reaction is with the aromatic ring. All the simple aromatic molecules with electron donating substituent have *k*_Cl•_ values above 1×10^10^ mol^–1^ dm^3^ s^–1^.

**Table 6:** Antibiotics

Compound, p*K*_a_	*k*_Cl•_, mol^–1^ dm^3^ s^–^^1^	Method, pH	Reference
6-Aminopenicillanic acid, 2.7	3.4 ± 0.3×10^9^	LFP,C, 7	Lei et al. 2019
Amoxicilin, 2.6, 7.2	1.27 ± 0.08×10^10^	LFP,C, 7	Lei et al. 2019
	7.9 ± 0.6×10^9^	LFP, 7	
Penicillin G, 2.9	1.25 ± 0.09×10^10^	LFP,C, 7	Lei et al. 2019
Penicillin V, 2.6	1.31 ± 0.09×10^10^	LFP,C, 7	Lei et al. 2019
7-Aminocephalosporanic acid, 2.6	1.14 ± 0.07×10^10^	LFP,C, 7	Lei et al. 2019
Cefotaxime, 2.7	2.30 ± 0.12×10^10^	LFP,C, 7	Lei et al. 2019
Cephalexin, 2.6, 6.4	2.17 ± 0.25×10^10^	LFP,C, 7	Lei et al. 2019
Cefaclor, 1.6	1.59 ± 0.20×10^10^	LFP,C, 7	Lei et al. 2019
Ciprofloxacin, 6.1, 8.7	1.39 ± 0.35×10^10^	LFP,C, 7	Lei et al. 2019
Enrofloxacin, 6.2, 7.6	1.53 ± 0.40×10^10^	LFP,C, 7	Lei et al. 2019
Ofloxacin, 5.7	1.54 ± 0.25×10^10^	LFP,C, 7	Lei et al. 2019
Flumequine, 6.5	7.7 ± 2.3×10^9^	LFP,C, 7	Lei et al. 2019
Azithromycin, 8.6	7.8 ± 0.4×10^9^	LFP,C, 7	Lei et al. 2019
	8.3 ± 0.3×10^9^	LFP, 7	
Erythromycin, 8.9	6.8 ± 0.3×10^9^	LFP,C, 7	Lei et al. 2019
	7.0 ± 0.3×10^9^	LFP, 7	
Roxithromycin, 9.2	7.2 ± 0.7×10^9^	LFP,C, 7	Lei et al. 2019
Dimetridazole, 2.8	4.2 ± 0.3×10^9^	LFP,C, 7	Lei et al. 2019
Metronidazole, 2.6	5.64 ± 0.1 × 10^9^	Comp., 7	Pan et al. 2019
	3.1 ± 0.5×10^9^	LFP,C, 7	Lei et al. 2019
Sulfanilamide, 2.0, 5.3	3.12 ± 0.40×10^10^	LFP,C, 7	Lei et al. 2019
Sulfadimethoxine, 2.9, 6.1	4.08 ± 0.24×10^10^	LFP,C, 7	Lei et al. 2019
Sulfadiazine, 2.1, 6.4	3.35 ± 0.22×10^10^	LFP,C, 7	Lei et al. 2019
Sulfamethazine, 2.6, 7.4	3.21 ± 0.11×10^10^	LFP,C, 7	Lei et al. 2019
Sulfamethoxazole, 1.6, 5.7	4.4–5.4×10^9^	Est	Li et al. 2017
	3.4 ± 0.4×10^10^	Comp. 7	Sun et al. 2019
	3.64 ± 0.21×10^10^	LFP,C, 7	Lei et al. 2019
Sulfathiazole, 2.2, 7.2	3.78 ± 0.49×10^10^	LFP,C, 7	Lei et al. 2019
Sulfapyridine, 8.43	8.79 ± 0.27×10^9^	Compl., 5	Liu et al. 2020
2-Phenylbenzimidazole-5-sulfonic acid	1.5×10^10^	Comp., 7	Yin et al. 2022
Oxytetracycline, 3.1, 7.4, 8.9	2.36 ± 0.56×10^10^	LFP,C, 7	Lei et al. 2019
Tetracycline, 3.32, 7.78, 9.58	1.98 ± 0.42×10^10^	LFP,C, 7	Lei et al. 2019
Trimethoprim, 3.2, 7.12	1.9×10^10^	Est	Wu et al. 2016
	2.11 ± 0.12×10^10^	LFP,C, 7	Lei et al. 2019
	1.69 ± 0.63×10^10^	LFP, 7	
Trimethoprim, diion	6.52×10^9^	Fit	Wang et al. 2021a
Trimethoprim, monoion	3.09×10^9^	Fit	
Trimethoprim, neutral	7.76×10^9^	Fit	

7-Aminocephalosporanic acid is regarded as the structural unit, which carries the antibacterial potency of the cephalosporin type β-lactam antibiotics. Its *k*_Cl•_ value, 1.14 ± 0.07×10^10^ mol^–1^ dm^3^ s^–1^, is close to those of the cephalosporins in Table 6, cefotaxime, cephalexin and cefaclor, ~2×10^10^ mol^–1^ dm^3^ s^–1^ (Lei et al. 2019). This closeness of the values shows that in cephalosporins the β-lactam part is an important reaction centre in Cl^•^ reaction.

The quinolone antibiotics listed in Table 6 are used in cases of a large number of bacterial infections. Ciprofloxacin, enrofloxacin and ofloxacin have piperazine ring in their structures, they react with Cl with rate constant of ~1.5×10^10^ mol^–1^ dm^3^ s^–1^ (Lei et al. 2019). The rate constant measured for flumequine, which does not have piperazine ring, is only half of that value (7.7 ± 2.3×10^9^ mol^–1^ dm^3^ s^–1^, Lei et al. 2019). The piperazine ring may show high reactivity toward Cl^•^.

In the macrolide type antibiotics (azithromycin, erythromycin, roxithromycin), there is a 14 membered macrolide ring, two sugar molecules are linked to this ring. These molecules do not have double bonds or aromatic rings in their structures, susceptible site for Cl^•^ attack. Although these are large molecules, the rate constants are relatively small, for all of them values around 7.5×10^9^ mol^–1^ dm^3^ s^–1^ were published (Lei et al. 2019). For the reactions of dimetridazole and metronidazole, rate constants of ~4.5×10^9^ mol^–1^ dm^3^ s^–1^ were published (Pan et al. 2019; Lei et al. 2019). The similar rate constants for the two nitroimidazole antibiotics suggest that the main site of Cl^•^ attack is the nitroimidazole ring.

The rate constants for the sulfa drugs in Table 6 (sulfanilamide, sulfadimethoxine, sulfadiazine, sulfamethazine, sulfamethoxazole and sulfathiazole) are high; they are in the 3×10^10^–4×10^10^ mol^–1^ dm^3^ s^–1^ range (Lei et al. 2019). Sulfapyridine represents an exception, the rate constant is somewhat smaller 8.79 ± 0.27×10^9^ mol^–1^ dm^3^ s^–1^. The latter measurement was made by Liu et al. (2020) by the competitive technique, they also accepted that nitrobenzene does not react with Cl^•^. At neutral pH, these antibiotics exist in neutral or in anionic forms. In hydroxyl radical reactions, also highly similar rate constants, close to the diffusion controlled limit, were established for all sulfonamides (Wojnárovits et al. 2018). In ^•^OH reaction, detailed final product studies were also conducted with general conclusion that ^•^OH reacts with both the sulfonamide and heterocyclic parts of these molecules. By analogy, Cl^•^ may also react in similar way. Liu et al. (2020) assumed that Cl^•^ mainly attacks the aniline part of these molecules.

2-Phenylbenzimidazole-5-sulfonic acid is a personal care product that is used to protect skin from damage upon solar irradiation. Its chemical structure is similar to those of sulfa drugs. In complex reaction kinetics system, using competitive kinetics, a rate constant of 1.5×10^10^ mol^–1^ dm^3^ s^–1^ is suggested for its reaction with Cl^•^ (Yin et al. 2022).

Tetracyclines have four hydrocarbon rings in their structures. They are relatively cheap antibiotics used both in human and animal therapy and at subtherapeutic levels as animal growth promoters. The rate constants of both tetracyclines in the table, tetracycline and oxytetracycline are close to the diffusion controlled *k*_Cl•_.

Trimethoprim as antibiotic mainly used in cases of urinary infections. This medicine is often used in combination with sulfa drugs, e.g., sulfamethoxazole or sulfadiazine. At low pH, both N-atoms in the heterocyclic ring are protonated, at high pH the neutral form dominates (p*K*_a1_ 3.1, p*K*_a2_ 7.1). Under the usual conditions, there is a pH dictated equilibrium between the dication (Trim^2+^), monocation (Trim^+^) and neutral forms (Wang et al. 2021a). Lei et al. (2019) based on direct and indirect (competitive) measurements at pH 7 and Wu et al. (2016) by estimation suggested *k*_Cl•_ ≈ 2×10^10^ mol^–1^ dm^3^ s^–1^. The value agrees with the *k*_Cl•_ of 1,3,5-timethoxybenzene, ~2×10^10^ mol^–1^ dm^3^ s^–1^, this molecule also has three methoxy groups on the benzene ring. Wang et al. (2021a) using the experimental degradation data in the UV/chlorine process and a complex kinetic system in their fitting procedure published, 6.52×10^9^, 3.09×10^9^ and 7.76×10^9^ mol^–1^ dm^3^ s^–1^ rate constant values for the dication, monocation and the neutral molecule, respectively. These values are much smaller than those determined by the previously mentioned authors. We have the feeling that the applied fitting procedure, due to the large number of reactions involved in the reaction system, and the uncertainties in the literature rate constants may have given only an order of magnitude estimate.

### Miscellaneous organic compounds

**Scheme 7.**
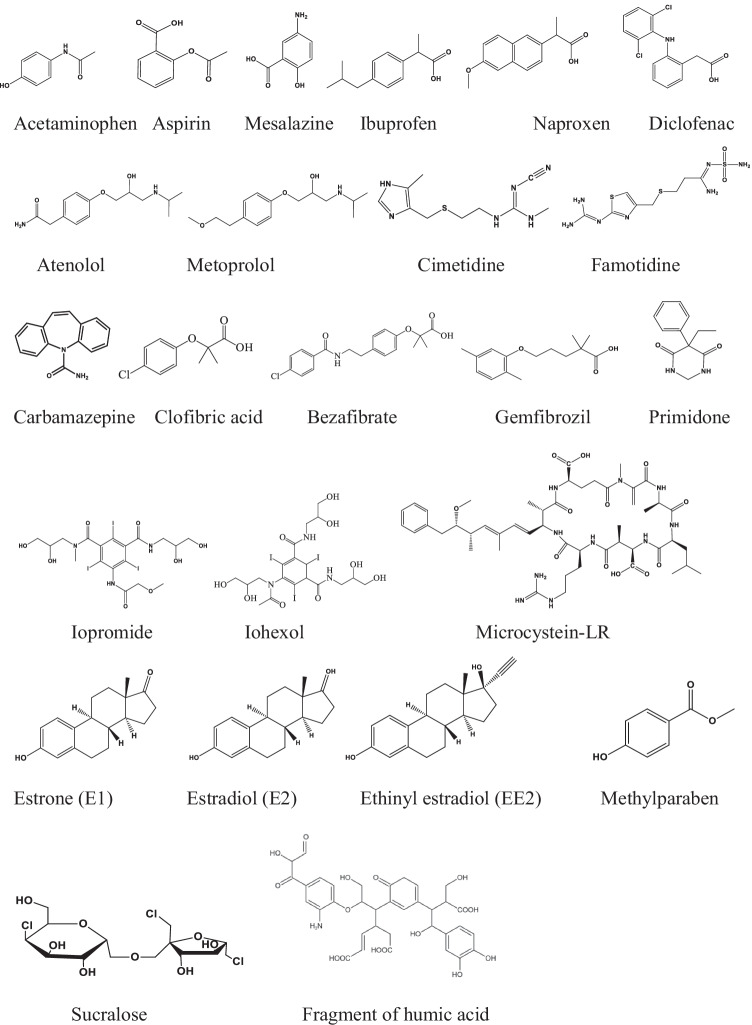
Miscellaneous molecules

The non-steroidal anti-inflammatory drugs, acetaminophen (paracetamol), aspirin (acetylsalicylic acid), mesalazine (5-aminosalicylic acid), ibuprofen, naproxen and diclofenac (Scheme 7, Table 7) all contain aromatic ring in their structures, a probable part of Cl^•^ attack. The measured rate constants, except for aspirin, are high; they are in the 1×10^10^–3×10^10^ mol^−1^ dm^3^ s^−1^ range (Giang et al. 2017; Lei et al. 2019; Li et al. 2020). In a recent paper, by calculations using transient state theory Wang et al. (2021b) published a lower value (2.61×10^9^ mol^−1^ dm^3^ s^−1^) for acetaminophen. This *k*_Cl•_ seems to be unrealistic in view of the measured values, and in view of the high rate constant values published for similar compounds (e.g., simple aromatic molecules). Aspirin has a *k*_Cl•_ of 6.8 ± 1.4×10^9^ mol^−1^ dm^3^ s^−1^. In this molecule, the acetyl group strongly decreases the electron density on the ring. The published rate constant of diclofenac reaction seems to be too high, 3.77 ± 0.65×10^10^ mol^−1^ dm^3^ s^−1^ (Lei et al. 2019) in view of the two electron withdrawing Cl atoms on one of the rings. However, it should be mentioned that high rate constant at the diffusion-controlled level was also experienced in the reaction of the other strong one-electron oxidant sulfate radical anion (Mahdi Ahmed et al. 2012).

**Table 7:** Rate constants of Cl.^•^ with miscellaneous organic compounds

Compound, p*K*_a_	*k*_Cl•_, mol^–1^ dm^3^ s^–^^1^	Method, pH	Reference
Acetaminophen (paracetamol), 9.4	3.71×10^10^	Comp., 5.5	Giang et al. 2017
	1.33 ± 0.19×10^10^	LFP,C, 7	Lei et al. 2019
	1.24 ± 0.26×10^10^	LFP,7	
	1.08×10^10^	Comp. 7	Li et al. 2020
	2.61×10^9^	Calc	Wang et al. 2021b
Aspirin, 3.4	6.8 ± 1.4×10^9^	LFP,C, 7	Lei et al. 2019
Mesalazine, 2.7, 5.8	2.23 ± 0.25×10^10^	LFP,C, 7	Lei et al. 2019
Ibuprofen, 4.9	2.77 ± 0.35×10^10^	LFP,C, 7	Lei et al. 2019
	1.3 ± 0.2×10^10^	LFP, 3.9	Wu et al. 2019
Naproxen, 4.2	2.01 ± 0.15×10^10^	LFP,C, 7	Lei et al. 2019
	2.7 ± 0.3×10^10^	LFP, 3.9	Wu et al. 2019
	4.9×10^9^	Compl	Liu et al. 2021
Diclofenac, 4.15	3.77 ± 0.65×10^10^	LFP,C, 7	Lei et al. 2019
Atenolol, 9.5	1.12×10^9^	Comp., 5.8	Mangalgiri et al. 2019
	2.29 ± 0.23×10^10^	LFP,C, 7	Lei et al. 2019
Metoprolol	1.71 ± 0.31×10^10^	LFP,C, 7	Lei et al. 2019
Cimetidine, 7.1	4.3 ± 1.1×10^9^	LFP,C, 7	Lei et al. 2019
Famotidine, 1.8, 6.8	1.72 ± 0.26×10^10^	LFP,C, 7	Lei et al. 2019
Carbamazepine, 13.9	5.6 ± 1.6×10^10^	Compl., 7	Wang et al. 2016
	1.8–3.7×10^9^	Est	Li et al. 2017
	3.7 ± 0.3×10^10^	Comp., 7	Sun et al. 2019
	3.30 ± 0.26×10^10^	LFP,C, 7	Lei et al. 2019
	1.84×10^9^	Fit	Zhu et al. 2021
Clofibric acid, 3.18	9.76 ± 0.15 × 10^10^	Comp., 7	Lu et al. 2018
	5.5 ± 1.3×10^9^	LFP,C, 7	Lei et al. 2019
Bezafibrate, 3.6	5.0×10^8^	Est	Shi et al. 2018
	1.04 ± 0.09×10^10^	LFP,C, 7	Lei et al. 2019
Gemfibrozil, 4.5	2.14 ± 0.17×10^10^	LFP,C, 7	Lei et al. 2019
	1.2×10^9^	Compl	Liu et al. 2021
	1.4×10.^10^	LFP, 7	Chen et al. 2021
Primidone, 12.3	6.2 ± 1.0×10^9^	LFP,C, 7	Lei et al. 2019
	3.19×10^10^	Comp, 5	Wang et al. 2020
Iopromide, 10.6	2.75 ± 0.39×10^10^	LFP,C., 7	Lei et al. 2019
Iohexol, 11.7	1.18 ± 0.22×10^12^	Compl	Zhu et al. 2019
Microcystein-LR, 3.0	2.25 ± 0.07×10^10^	LFP, 3.9	Zhang et al. 2019
Estrone (E1), 10.7	2.06 ± 0.21×10^10^	LFP,C, 7	Lei et al. 2019
Estradiol (E2), 10.4	1.3–1.6×10^10^	Est	Li et al. 2017
	8.0 ± 0.2×10^9^	Comp.7	Sun et al. 2019
	2.01 ± 0.30×10^10^	LFP,C, 7	Lei et al. 2019
Ethinyl estradiol (EE2), 10.7	2.1 ± 0.2×10^9^	Comp., 7	Sun et al. 2019
	2.56 ± 0.11×10^10^	LFP,C, 7	Lei et al. 2019
Methylparaben, 8.3	1.52 ± 0.13×10^10^	LFP,C, 7	Lei et al. 2019
Sucralose, 12.5	1.11 ± 0.16×10^10^	LFP,C, 7	Lei et al. 2019
Humic acid	3 ± 2×10^10^	FP, 4	Caregnato et al. 2007

The reactivity of Cl^•^ with atenolol and metoprolol (β blockers) is similar to that of ^•^OH and the basic reaction mechanism is radical addition to the double bonds. Cl^•^ reacts with atenolol and metoprolol with rate constant above 1×10^10^ mol^−1^ dm^3^ s^−1^ (Mangalgiri et al. 2019; Lei et al. 2019).

Cimitidine and famotidine are used to control stomach acid overproduction. They contain a sulfur atom in the alkyl chain. Based on analogous reactions this S bridge is expected to be the main target in one-electron oxidation (Wojnárovits and Takács 2021). The Cl^•^ rate constants of the two compounds are highly different: 4.3 ± 1.1×10^9^ (cimetidine) and 1.72 ± 0.26×10^10^ mol^−1^ dm^3^ s^−1^ (famotidine, Lei et al. 2019). Based on the highly different values, we assume that the main place of Cl^•^ attack is not the S-atom, since its surrounding in the chain is the same in both molecules.

For the rate constant of carbamazepine, Wang et al. (2016) published an unrealistically high value of 5.6 ± 1.6×10^10^ mol^–1^ dm^3^ s^–1^ based on competitive experiments in the complicated carbamazepine-nitrobenzene-benzoic acid system. Under their conditions, both ^•^OH and Cl^•^ reacted with the solutes. Sun et al. (2019) also used the assumption of negligible reaction between Cl^•^ and nitrobenzene and their rate constant value is also high (3.7 ± 0.3×10^10^ mol^–1^ dm^3^ s^–1^). Lei et al. (2019) in laser flash photolysis experiments found a just bit smaller value, 3.30 ± 0.26×10^10^ mol^–1^ dm^3^ s^–1^. Li et al. (2017) using rate constant values on similar compounds estimated unrealistically small *k*_Cl•_ of 1.8–3.7×10^9^ mol^–1^ dm^3^ s^–1^.

Clofibric acid (metabolite of several lipid regulators) has a similar reactive part (Cl-Ph-O-R) as climbazole. Lei et al. (2019) published a rate constant of 5.5 ± 1.3×10^9^ mol^−1^ dm^3^ s^−1^ using the SCN^−^ competitive technique in laser flash photolysis experiments for the Cl^•^ + clofibric acid reaction. For this reaction, Lu et al. (2018) published unrealistically high value, 9.76 ± 0.15×10^10^ mol^−1^ dm^3^ s^−1^. They also used nitrobenzene as probe molecule, assuming that its reaction with Cl^•^ was negligible. The unrealistically high rate constant here also demonstrates that this technique supplies false results. Bezafibrate and gemfibrozil both contain some fragment of clofibric acid. In the former one, there are two aromatic rings (Cl is attached to one of them), in the latter there is one (and no Cl-atom is attached to the aromatic ring), giving explanation for the high rate constants: 1.04 ± 0.09×10^10^ and 2.14 ± 0.17×10^10^ mol^−1^ dm^3^ s^−1^, respectively (Lei et al. 2019). Lei et al. (2019) explained the high difference between the rate constants of clofibric acid and gemfibrozil in terms of the presence of two methyl groups on the benzene ring of gemfibrozil and the electron withdrawing chlorine atom on the benzene ring of clofibric acid. The *k*_Cl•_ value of Liu et al. (2021) for gemfibrozil (1.2×10^9^ mol^−1^ dm^3^ s^−1^) obtained in complex kinetic system is unrealistically low compared to the rate constants of compounds with similar structures.

Primidone is an epilepsy medicine. Lei et al. (2019) and Wang et al. (2020) determined highly different *k*_Cl•_ values, 6.2 ± 1.0 × 10^9^ and 3.19 × 10^10^ mol^−1^ dm^3^ s^−1^, respectively. The latter *k*_Cl•_ was also obtained using nitrobenzene probe molecule.

Due to the three heavy iodine atoms in their structures, iopromide and iohexol are used as X-ray contrast materials in the medical practice. *k*_Cl•_ published for iopromide is high, 2.75±0.39×10^10^ mol^−1^ dm^3^ s^−1^ (Lei et al. 2019). The value given for iohexol by Zhu et al. (2019) based on modeling calculations is completely unrealistic (1.18 ± 0.22×10^12^ mol^−1^ dm^3^ s^−1^), it is two orders of magnitude higher than the diffusion controlled limit.

Estrone and estradiol are natural hormones while ethinylestradiol (EE) is an estrogen medication which is widely used in birth control pills. Since estrogens are regularly detected in wastewaters and in natural waters, the mentioned compounds were often used as models in the UV/chlorine process. The rate constants measured by Lei et al. (2019) for the three compounds are ~2.2×10^10^ mol^–1^ dm^3^ s^–1^; the estimated value of Li et al. (2017) for estradiol (1.3×10^10^–1.6×10^10^ mol^–1^ dm^3^ s^–1^) does not differ much from the measured rate constant. However, the rate constant, 2.1 ± 0.2×10^9^ mol^–1^ dm^3^ s^–1^, suggested by Sun et al. (2019) for ethinyl estradiol is certainly unrealistically small. Since the basic structure of the three molecules is similar, we expect not much different rate constants.

Methylparaben (*p*-hydroxybenzoic acid methyl ester) is an anti-fungal agent often used in a variety of cosmetics and personal-care products. It is also used as a food preservative. The rate constant published for this molecule, 1.52 ± 0.13×10^10^ mol^–1^ dm^3^ s^–1^ (Lei et al. 2019) is close to the values measured for similar aromatic molecules, e.g., benzoic acid (Table 4). Sucralose is an often used artificial sweetener. Cl^•^ is expected to react with it in H-abstraction reaction: *k*_Cl•_ = 1.11 ± 0.16×10^10^ mol^–1^ dm^3^ s^–1^ (Lei et al. 2019).

## Discussion

### Reliability of the methods and the rate constant values

Previously 10–15 different techniques were mentioned used for rate constant determination. For several compounds (e.g., ethanol, *tert*-butanol, chloroacetone, estradiol, trimethoprim, acetaminophen, naproxen, carbamazepine), two or more rate constants were published eventually obtained in different laboratories by different methods. This fact allows us to say something about the reliability of the applied methods and/or about the obtained rate constant values.

In liquid phase reactions, the rate constants are limited by the diffusion. Buxton et al. (2000) suggested a diffusion controlled rate constant for Cl^•^ reactions around 8.5×10^9^ mol^−1^ dm^3^ s^−1^. Minakata et al. (2017) for the reactions of Cl^•^ with some inorganic ions calculated *k*_diff_ values of 1.1×10^10^ mol^−1^ dm^3^ s^−1^. Lei et al. (2019) in evaluation of their measured values considered a value of ~2×10^10^ mol^−1^ dm^3^ s^−1^. This value is between the *k*_diff_ values suggested for ^•^OH an H^•^: 1.1×10^10^ and 2.9×10^10^ mol^−1^ dm^3^ s^−1^, respectively (Wojnárovits and Takács 2016), and we tend to accept this rate constant as the diffusion controlled limit. Many of the published rate constants approach or even exceed 2×10^10^ mol^−1^ dm^3^ s^−1^. We suggest to disregard all the rate constants that are much (several times) higher than this value. Such as the rate constant of Wang et al. (2017) for carbamazepine (5.6±1.6 × 10^10^ mol^−1^ dm^3^ s^−1^) established in calculations using complex kinetic systems. The rate constant in the work of Zhu et al. (2019) on the iohexol reaction (1.18±0.22 × 10^12^ mol^−1^ dm^3^ s^−^) obtained in modeling calculations is completely unrealistic. Lu et al. (2018) and Cai et al. (2019) used the steady-state competitive technique to determine the *k*_Cl•_ of the clofibric acid and climbazole reactions (9.76±0.15×10^10^ and 6.3×10^10^ mol^−1^ dm^3^ s^−1^, respectively). They assumed negligible reaction between applied probe molecule nitrobenzene and Cl^•^, probably this is the reason of the unrealistically high values. The unrealistic values raise a question on the reliability of the applied techniques.

The values measured by Lei et al. (2019) (SCN^–^ competitive technique, LFP,C) for sulfonamides, 3.12×10^10^–4.08×10^10^ mol^−1^ dm^3^ s^−1^, xanthines 3.81×10^10^–3.98×10^10^ mol^−1^ dm^3^ s^−1^, diclofenac 3.77×10^10^ mol^−1^ dm^3^ s^−1^ are also examples of the high values. These authors mention a possible explanation: Cl^•^ may react with these molecules via single electron transfer (SET) reaction, by which the two reactants do not necessarily need to diffuse and encounter in the solvent cage.

In some cases, by using quantum chemical calculations, complex fitting, or modeling procedures too small values were given. For example: the average of four values published for acetaminophen using competitive or product build-up experiments is 1.8 × 10^10^ mol^−1^ dm^3^ s^−1^ (Giang et al. 2017; Lei et al. 2019; Li et al. 2020), Wang et al. (2021b) based on their calculations reported an order of magnitude smaller value, 2.61×10^9^ mol^−1^ dm^3^ s^−1^. Zhu et al. (2021) gave an order of magnitude lower value for the carbamazepine reaction also as established by other authors.

It is obvious that one has to be rather careful when using a technique for rate constant determination. Especially much care should be taken when Cl^•^ produced in the UV/chlorine process is used in combination with simulation, complex fitting or with competitive (nitrobenzene problem) techniques.

### Reaction mechanisms

Very few mechanistic studies were conducted in connection with the Cl^•^ reaction to determine the elementary reaction steps of radical attack on organic molecules, e.g., on identifying the primary organic intermediates. This is because many rate constant determinations were made under steady-state conditions (e.g., using the UV/chlorine processes) which give no possibilities to observe the elementary processes. In the transient kinetic experiments (pulse radiolysis, (laser) flash photolysis) the absorbances of Cl^•^ and Cl_2_^•–^ complicate the observations of transient organic intermediates (see in the Introduction). However, there are important exceptions, such as 1,3,5-trimethoxybenzene (TMB). In the low pH range, the reaction with Cl^•^ gives radical cation (TMB^•+^):

28 $${\mathrm{Cl}}^{\bullet }+\mathrm{TMB} \to {\mathrm{Cl}}^{-}+{\mathrm{TMB}}^{\bullet +}$$

This radical cation has a p*K*_a_ value between pH 3.0 and 5.0 and exhibits strong transient absorbance with *λ*_max_ = 580 nm (Lei et al. 2019). Based on the intensity of the absorbance, the authors estimated a single-electron-transfer (SET) contribution of 62.4% to the Cl^•^ + TMB reaction. SET also had important contributions to the degradation of some other molecules, such as the sulfonamides. In the transient spectra of these molecules, strong absorptions were observed in the 400–440 nm range, which were attributed to the formation of aniline type radical cations (C_6_H_5_NH_3_^•+^). Reaction with SET mechanism is suggested also in the reactions of several inorganic ions and alcohols with Cl^•^ (Gilbert et al. 1988). SET reaction pathway was disregarded in the cases of benzene, toluene and benzoic acid, since the expected final products in this mechanism, the phenol derivatives were not observed (Mártire et al. 2001).

The most often suggested reaction between Cl^•^ and organic molecules with unsaturated bonds including also aromatics is the radical addition to the double bond (Minakata et al. 2017). This reaction is similar to the reaction of ^•^OH, but Cl^•^ reactions show less selectivity as those of ^•^OH. Cl^•^ addition to the aromatic ring yields chlorocyclohexadienyl radicals with absorption bands in the 300–350 nm range (Alegre et al. 2000; Mártire et al. 2001; Lei et al. 2019). The aqueous medium seems to stabilize the radical adducts and they decay on much longer timescale as Cl^•^. This stabilization does not exist in the gas phase (Mártire et al. 2001). In contrast to the non-aqueous systems π-complex formation was not observed in aqueous solutions.

Hydrogen atom abstraction is typical reaction of molecules without double bonds. In the reaction a definite bond-strength effect is observed.

## Summary

Rate constants of Cl^•^ reactions were collected from the literature and were discussed together with the methods of determinations and the reaction mechanisms. These rate constants were determined using a large variation of experimental techniques including transient measurements of Cl^•^ decay at *λ*_max_ = 320 nm (when the absorbances of the starting molecules and the short-lived products do not disturb the observation), or of build-up of the product intermediate at longer wavelengths. Large number of measurements were made in laser flash photolysis experiments utilizing the competition with SCN^–^ ions. In another group of experiments, the rate constants were derived from rather complex reaction systems considering a large number of elementary reactions, using simulations/modeling or fitting procedures and often involving in the determinations radical scavenging experiments. The latter experiments often gave unrealistic rate constant values.

The rate constant values are generally in the 10^8^–10^9^ mol^−1^ dm^3^ s^−1^ range when the basic reaction between Cl^•^ and the target molecule is H-atom abstraction. When the Cl^•^ atom addition to a double bonds dominate the interaction the rate constants are in the 1× 10^9^–2×10^10^ mol^−1^ dm^3^ s^−1^ range. In the *k*_Cl•_ = 1×10^10^–4×10^10^ mol^−1^ dm^3^ s^−1^ range single-electron-transfer reactions may also contribute to the mechanism.

The reactions of Cl^•^ with organic molecules in many respects are similar to the reactions ^•^OH, albeit the chlorine atom seems to be less selective as the hydroxyl radical. However, there is an important difference; in case of ^•^OH single-electron-transfer reactions have minor importance.

Since Cl^•^ atom reactions play very important role in the emerging UV/chlorine technology, some standardization of the rate constant measuring techniques and more *k*_Cl•_ measurements are needed.

## Data Availability

Data sharing is not applicable to this article as no datasets were generated or analyzed during the current study.
